# Association of Biomarkers for Human Papillomavirus With Survival Among Adults With Barrett High-grade Dysplasia and Esophageal Adenocarcinoma

**DOI:** 10.1001/jamanetworkopen.2019.21189

**Published:** 2020-02-14

**Authors:** Shanmugarajah Rajendra, Preeti Sharma, Shweta Dutta Gautam, Manoj Saxena, Amit Kapur, Prateek Sharma, Neil Merrett, Tao Yang, Leonardo D. Santos, Darren Pavey, Omar Sharaiha, Owen McKay, Hugh Dixson, Wei Xuan

**Affiliations:** 1 Gastro-Intestinal Viral Oncology Group, Ingham Institute for Applied Medical Research, Liverpool, Sydney, New South Wales, Australia; 2 South Western Sydney Clinical School, University of New South Wales, Liverpool, Sydney, New South Wales, Australia; 3 Department of Gastroenterology and Hepatology, Bankstown-Lidcombe Hospital, South Western Sydney Local Health Network, Bankstown, Sydney, New South Wales, Australia; 4 Department of Intensive Care, Bankstown-Lidcombe Hospital, South Western Sydney Local Health Network, Bankstown, Sydney, New South Wales, Australia; 5 Graduate School of Medicine, The University of Wollongong, Wollongong, New South Wales, Australia; 6 Veterans Affairs Medical Center, Division of Gastroenterology and Hepatology, University of Kansas School of Medicine, Kansas City, Missouri; 7 Discipline of Surgery, Western Sydney University School of Medicine, Penrith, New South Wales, Australia; 8 Department of Upper Gastrointestinal Surgery, Bankstown-Lidcombe Hospital, Bankstown, Sydney, New South Wales, Australia; 9 SydPath, St Vincent’s Hospital Sydney, Darlinghurst, New South Wales, Australia; 10 Department of Anatomical Pathology, Sydney South West Pathology Service, Liverpool Hospital, Liverpool, Sydney, New South Wales, Australia; 11 Department of Nuclear Medicine, Bankstown-Lidcombe Hospital, South Western Sydney Local Health Network, Bankstown, Sydney, New South Wales, Australia; 12 Ingham Institute for Applied Medical Research, Liverpool, Sydney, New South Wales, Australia

## Abstract

**Importance:**

The presence of high-risk human papillomavirus (HPV) has been associated with a favorable outcome in Barrett high-grade dysplasia (HGD) and esophageal adenocarcinoma (EAC). Nevertheless, the prognostic significance of other HPV-related biomarkers (ie, retinoblastoma protein [pRb], cyclin D1 [CD1], minichromosome maintenance protein [MCM2] and Ki-67) is unknown.

**Objective:**

To examine the association between HPV-related biomarkers and survival in adult patients with Barrett HGD and EAC.

**Design, Setting, and Participants:**

This retrospective case-control study examined the hypothesis that the HPV-related cell cycle markers (pRb, CD1, and Ki-67) and the viral surrogate marker (MCM2) may be associated with a favorable prognosis in Barrett HGD and EAC. Pretreatment biopsies were used for HPV DNA determination via polymerase chain reaction and immunohistochemistry for the HPV-related biomarkers. Recruitment of patients occurred in secondary and tertiary referral centers, with 151 patients assessed for eligibility. The study period was from December 1, 2002, to November 28, 2017, and the dates of analysis were from September 9, 2011, to November 28, 2017.

**Main Outcomes and Measures:**

Disease-free survival and overall survival.

**Results:**

Of 151 patients assessed for eligibility, 9 were excluded. Among the 142 patients with Barrett HGD or EAC (126 [88.7%] men; mean [SD] age, 66.0 [12.1] years; 142 [100%] white), 37 were HPV positive and 105 were HPV negative. No association with disease-free survival was noted for pRb, CD1, Ki-67, and MCM2. In regard to overall survival, only low expression of CD1 had a favorable prognosis (hazard ratio [HR], 0.53; 95% CI, 0.30-0.95; adjusted *P* = .03). All the biomarkers stratified by HPV status showed significant associations with survival. Patients with HPV-positive, low-expression pRb esophageal tumors were associated with a significantly improved disease-free survival compared with the HPV-negative, high-expression Rb tumors (HR, 0.33; 95% CI, 0.12-0.93; adjusted *P* = .04). Similarly, HPV-positive, low-expression CD1 was associated with a significantly favorable disease-free survival (HR, 0.26; 95% CI, 0.09-0.76; adjusted *P* = .01), as was HPV-positive, high-expression MCM2 (HR, 0.27; 95% CI, 0.09-0.78; adjusted *P* = .02). In regard to overall survival, HPV was significantly associated only with low CD1 (HR, 0.38; 95% CI, 0.15-0.94; adjusted *P* = .04).

**Conclusions and Relevance:**

This study’s findings suggest that low expression of CD1 appears to be an independent prognostic marker in Barrett HGD and EAC. Human papillomavirus positivity in combination with pRb, CD1, MCM2, and Ki-67 was associated with a survival benefit in esophageal tumors. These findings suggest the possibility of personalization of therapy for Barrett HGD and EAC based on viral status.

## Key Points

**Question:**

What is the prognostic significance of human papillomavirus–related biomarkers (ie, retinoblastoma protein, cyclin D1, minichromosome maintenance protein, and Ki-67) in Barrett high-grade dysplasia and esophageal adenocarcinoma?

**Findings:**

In this case-control study of 142 patients with Barrett high-grade dysplasia and esophageal adenocarcinoma, only low cyclin D1 levels were associated with a favorable prognosis for overall survival. None of the biomarkers tested independently had any association with disease-free survival: pRb, CD1, MCM2, and Ki-67 stratified by human papillomavirus status was associated with a survival benefit in esophageal tumors.

**Meaning:**

These findings suggest the possibility of personalization of therapy for Barrett high-grade dysplasia and esophageal adenocarcinoma based on human papillomavirus status.

## Teaser

This case-control study examines whether high-risk human papillomavirus–related biomarkers are associated with survival among adult patients with Barrett high-grade dysplasia and esophageal adenocarcinoma.

## Introduction

Transcriptionally active high-risk-human papillomavirus (HPV) infection has been associated with Barrett dysplasia and esophageal adenocarcinoma (EAC).^1,2^ Nevertheless, studies showing no association with HPV and Barrett dysplasia and EAC exist. Reasons for the negative findings include poor tissue classification, suboptimal testing methods, small sample sizes, racial and geographic variations, and the use of metaplastic tissue that is not associated with the virus.^3,4,5,6,7^ However, a systematic review published before these studies reported HPV prevalence rates of 35% in 174 patients with EAC.^8^ Another systematic review that included 19 studies found that the pooled prevalence of HPV in EAC was 13%. The authors suggested that the low prevalence rate may have been caused by small sample sizes and compromised detection methods.^9^

Human papilloma virus–positive Barrett high-grade dysplasia (HGD) and EAC seem to be distinct biological entities with a favorable prognosis compared with HPV-negative esophageal tumors and may benefit from treatment deescalation.^10,11^ A previous study demonstrated superior disease-free survival for HPV, transcriptionally active virus, E6 and E7 messenger RNA (mRNA), and high p16 expression, but not p53.^11^ Likewise, HPV-induced head and neck squamous cell carcinomas are a distinct subset with a more favorable prognosis compared with HPV-negative oropharyngeal cancers.^12,13^

Transcriptionally active HPV (DNA positive determined by polymerase chain reaction [PCR] and the presence of ≥1 of 2 markers of biological activity, ie, E6/E7 mRNA and/or p16^INK4A^) involvement in Barrett dysplasia and EAC is characterized by wild-type p53 and aberrations of the retinoblastoma protein (pRb) pathway (downregulation of the pRb as well as upregulation of p16^INK4A^).^14^ We investigated the prognostic significance of other cell-cycle markers, in particular, those related to HPV: pRb, CD1, minichromosome maintenance protein (MCM2), and Ki-67. Retinoblastoma protein, MCM2, and Ki-67 are considered surrogate markers of HPV oncogene expression (E6/E7, which are 2 viral proteins required for malignant cell transformation in HPV-driven cancers) when present in the upper layers of cervical lesions.^15,16^ A combination of Ki-67 and HPV status has been demonstrated to provide apparently superior prognostic information compared with HPV status alone in HPV-induced head and neck squamous cell carcinomas.^17^ Ki-67 is a cellular marker for proliferation and is expressed during all active phases of the cell cycle (G1, S, G2, and M) but is absent in the resting G0 phase. The pRb is a tumor-suppressor protein that exerts negative (checkpoint) control of the cell cycle and reduces excessive growth; pRb is degraded by the viral E7 oncoprotein with downregulation of pRb and CD1 and upregulation of p16^INK4A^.^18,19,20^

Increased expression of CD1 (a protein required for progression through the G1 phase of the cell cycle) has been documented in EAC.^21,22^ An earlier study investigating a molecular signature in virally associated esophageal cancer and its precursor lesion reported no association between CD1 and HPV DNA-positive and mRNA-positive (predominantly low-grade) Barrett dysplasia and EAC.^14^ Nevertheless, we did not investigate the prognostic significance of CD1 in Barrett dysplasia and EAC irrespective of HPV status. There are conflicting reports on the use of CD1 as a marker for other HPV-associated lesions,^23,24^ such as head and neck cancers and cervical cancer.^25^

Minichromosome maintenance protein is a family of 6 related proteins (MCM2-MCM7) that are involved in the initiation of DNA replication. Increased levels of MCM indicate proliferation of malignant cells as expressed in several types of cancers and is possibly more accurate than Ki-67 as a marker of proliferation.^26,27^ Persistent expression of MCM2, MCM5, and Ki-67 may be diagnostic markers in Barrett dysplasia.^28^ Furthermore, MCMs can predict tumor progression and thus are prognostic markers. Significant expression of MCM2 has been reported in Barrett dysplasia and EAC and in Barrett esophagus at subsequent risk of disease progression.^29^ Moreover, patients with EAC who have greater than 70% expression levels for MCM4 (high expression) had reduced survival compared with those with less than or equal to 70% nuclear staining for MCM4 (low expression).^30^ Abnormal expression of MCM2 in HPV-associated cervical cancer and cervical intraepithelial neoplasia has resulted in its use as a screening test for these lesions.^31,32^

Given the differential mutational and molecular landscape between HPV-positive and HPV-negative esophageal dysplastic and adenocarcinoma lesions, we hypothesized that viral-related cell-cycle proteins (eg, CD1 and pRb) and surrogate markers of viral E6 and E7 oncogene expression (MCM2 and Ki-67) may vary in these 2 biologically distinct tumors.^10,14^ Furthermore, we investigated the association between HPV DNA status and the prognostic value, if any, of the aforementioned biomarkers to elucidate a potential interaction of the virus.

## Methods

### Study Population

In this retrospective case-control study, eligible patients were those with Barrett HGD or EAC undergoing treatment with endotherapy (endoscopic mucosal resection and/or radiofrequency ablation) or esophagectomy with or without neoadjuvant chemoradiotherapy as previously described.^11^ The enrollment period was from December 1, 2002, to November 28, 2017. Study institutions included a tertiary referral center (Bankstown-Lidcombe Hospital, Sydney, New South Wales, Australia; n = 139) and a regional health care center Launceston General Hospital, Launceston, Tasmania, Australia; n = 3). Demographic (age, sex, body mass index) and clinical (ever smoked, excess alcohol use, proton pump inhibitor use, and resection margin status) data were obtained from a prospectively maintained database. Inclusion and exclusion criteria have been previously documented.^14^ Staging was performed as per the 7th edition of the *AJCC Cancer Staging Manual* by the American Joint Committee on Cancer.^33^ Oral and written consent was obtained from participants prior to the investigation. This study was approved by the Human Research Ethics Committee, Tasmania and South Western Sydney Local Health Network. The participants did not receive financial compensation. This study followed the Strengthening the Reporting of Observational Studies in Epidemiology (STROBE) reporting guideline for case-control studies.

### Laboratory Studies

Detection of HPV in genomic DNA extracted from fresh-frozen or formalin-fixed biopsy tissue was performed by nested PCR amplification of a conserved viral L1 gene using MY09 and MY11 and GP5^+^ and GP6^+^ primers for both high-risk and low-risk HPV as previously published.^1^ To minimize contamination, separate rooms were used for reaction preparation, template handling, performing nested reactions, and post-PCR analysis. Routine decontamination by UV irradiation was performed in the DNA-free PCR hood before each run. To guard against systematic contamination of PCR reagent, appropriate positive (HPV16-positive cervical cancer) and negative (deionized water and PCR master mix without template) controls were included in each step of the PCR process. The HPV genotypes were determined by sequencing.^1^ Real-time PCR assays measuring HPV E6 and E7 copy numbers using genotype-specific HPV-16 and HPV-18 primers were used to ascertain viral load.^2^

Expression of pRb, CD1, MCM2, and Ki-67 was assessed by immunohistochemistry on formalin-fixed, paraffin-embedded tissue (EnVision FLEX Mini Kits; Dako). Expression of pRb and CD1 was evaluated as previously described.^14^ For both Ki-67 and MCM2 after pretreatment, antigen retrieval was carried out using a high pH target retrieval solution for 20 minutes in a 98 °C water bath. After cooling, endogenous peroxidase was blocked by peroxidase-blocking reagent; sections were then incubated with primary antibodies monoclonal mouse antihuman Ki-67 antigen (1:100, Clone MIB-1; Dako) and anti-MCM2 rabbit polyclonal antibody (1:100, ab31159; Abcam) for 20 minutes at room temperature. The sections were washed with 1X wash buffer and incubated with secondary antibody (FLEX/HRP; EnVision) for 20 minutes at room temperature. DAB substrate + chromogen was used for color development. Sections were counterstained with hematoxylin for better visualization of tissue morphologic characteristics. Negative control was included by substitution of primary and secondary antibodies with human serum. A tissue section of cancer known to be positive for the particular protein marker studied was included in each run. Normal esophageal squamous tissue from a lesion-free patient was used as a staining reference for all 4 cellular protein markers.^14^ The microtome blade was replaced with sectioning of each new specimen to prevent cross-contamination.

All immunohistochemical scoring of slides was independently performed by 2 experienced gastrointestinal pathologists (T.Y. and L.D.S.) blinded to the virologic status and clinical outcome of the patients. For ease of evaluation, only 2 categories of staining were applied to all of the biomarkers (ie, high or low expression). For pRb and CD1 nuclear staining of at least moderate intensity in a minimum of 25% of the esophageal lesional cells was considered high expression. Less than 25% staining of pRb and cyclin D1 was scored as low expression.^14,34^ For Ki-67, a nuclear staining percentage greater than 20% was considered increased proliferation. Less than 20% was scored as 0, 21% to 50% was considered moderate proliferation with a score of 1, and more than 50% was considered strong proliferation and scored as 2.^35^ For the purposes of this study, a score of 1 or 2 was considered Ki-67-positive and a score of 0 was considered Ki-67 negative. The MCM2 was scored 0 for lack of staining, 1 for up to 30% positive staining of the nucleus, and 2 for greater than 30% positive staining of the nucleus. Any diffuse nuclear staining greater than 30% was considered high expression and less than or equal to 30% as low expression.^28^

### Statistical Analysis

The primary end points were disease-free survival from the time of diagnosis to the date of the first failure (local, regional, or distant) and overall survival, defined as the time between diagnosis and the date of death or last follow-up. Differences between HPV-positive vs HPV-negative cases in regard to baseline characteristics were assessed using the 2-sample *t* test for comparing the mean values between the 2 groups in regard to all numeric data. The association between the binary measurements in the viral-positive and viral-negative groups was evaluated using χ^2^ analysis. Survival analysis was conducted using the Kaplan-Meier method to estimate the disease-free survival and overall survival of the combination of HPV status and the 4 biomarkers (ie, pRb, CD1, MCM2, and Ki-67). Cox proportional hazards regression models were used to estimate the importance of these variables for disease-free survival and overall survival after adjusting for age, sex, body mass index, ever smoked, excess alcohol use, proton pump inhibitor use, nonsteroidal anti-inflammatory drug use, statin use, and surgical or endoscopic mucosal resection margin status. Any interaction between HPV status and the 4 biomarkers was explored, followed by stratified analysis as appropriate. Laboratory and data analysis were performed from September 9, 2011, to November 28, 2017.

All statistical tests were performed using SAS software, version 9.4 (SAS Institute Inc), and the level of significance was set at *P* < .05.

## Results

### Patient Characteristics

One hundred fifty-one patients were assessed for eligibility; 9 were excluded because they had gastric carcinoma. Therefore, 142 individuals with Barrett HGD or EAC were included in the study. Of these, 126 patients (88.7%) were men and the mean (SD) age was 66.0 (12.1) years; all of the patients were white. The mean (SD) follow-up time was 33.4 (28.0) months (range, 2-159 months) for all patients and 43.8 (29.4) months (range, 3-159 months) for surviving patients. Among the 37 HPV DNA-positive lesions (HPV16, n = 33; HPV18, n = 1; HPV6, n = 1; and HPV11, n = 2), median viral load was 0.1 copy per 10-cell genomic DNA (0-1.12 copies per 10-cell genome). These data are depicted in eTable 1 in the Supplement and in a previous publication.^11^ There were no significant differences in treatment (ie, endotherapy, esophagectomy, chemotherapy, and radiotherapy) between patients with HPV-positive and HPV-negative Barrett HGD or EAC.

### Biomarker Status

All 142 patients were analyzed for HPV DNA, pRb, CD1, MCM2, and Ki-67 (eFigure 1 and eFigure 2 in the Supplement). Downregulation of pRb was present in 18 of 37 patients (48.6%) with HPV-positive Barrett HGD or EAC as opposed to 35 of 105 patients (33.3%) with HPV-negative Barrett HGD or EAC (*P* = .10) (eTable 1 in the Supplement). Similarly, low expression of CD1 was not statistically significantly different between HPV-positive (22 of 37 [59.5%]) and HPV-negative (54 of 105 [51.4%]) EAC (*P* = .40). Again, there was no appreciable difference in Ki-67 expression between HPV-positive (31 of 37 [83.8%]) and HPV-negative (79 of 105 [75.2%]) EAC (*P* = .28). In contrast, high MCM2 expression was significantly lower in HPV-positive Barrett HGD and EAC (15 of 37 [40.5%]) as opposed to HPV-negative (70 of 105 [66.7%]) (*P* = .005).

On univariate or multivariate analysis, none of the markers (low-expression pRb, low-expression CD1, Ki-67–positive, or high-expression MCM2) had any association with disease-free survival (Table 1). In regard to overall survival, only low expression of CD1 had a favorable prognosis even after adjusting for confounders (hazard ratio [HR], 0.53; 95% CI, 0.30-0.95; *P* = .03) (Table 1).

**Table 1.** Log-Rank and Multivariate Disease-Free Survival and Overall Survival Analysis

Characteristic	Disease-Free Survival				Overall Survival			
	Model 1^a^		Model 2^b^		Model 1^a^		Model 2^b^	
	HR (95% CI)	Unadjusted *P* Value	HR (95% CI)	Adjusted *P* Value	HR (95% CI)	Unadjusted *P* Value	HR (95% CI)	Adjusted *P* Value
Low pRb expression	0.85 (0.52-1.40)	.53	0.88 (0.52-1.49)	.64	1.22 (0.73-2.06)	.45	1.39 (0.79-2.43)	.25
Low CD1 expression	0.77 (0.48-1.23)	.28	0.75 (0.44-1.27)	.29	0.58 (0.34-0.97)	.04	0.53 (0.30-0.95)	.03
Ki-67–positive	0.73 (0.43-1.24)	.24	0.81 (0.45-1.46)	.48	0.90 (0.49-1.65)	.73	1.22 (0.61-2.46)	.58
High-expression MCM2	1.53 (0.93-2.52)	.10	1.37 (0.80-2.34)	.25	1.15 (0.68-1.94)	.61	1.04 (0.58-1.85)	.90

Abbreviations: CD1, cyclin D1; HR, hazard ratio; MCM, minichromosome maintenance protein; pRb, retinoblastoma protein.

^a^ Model 1 was a univariate analysis of each characteristic with disease-free survival.

^b^ In model 2, each characteristic was analyzed separately, using multivariate Cox proportional hazards regression, adjusted for the following covariates: age, sex, body mass index, ever smoked, excess alcohol use, proton pump inhibitor use, nonsteroidal anti-inflammatory drug use, statin use, and R0 resection margin.

### Biomarkers Stratified by HPV Status

**Figure 1.**
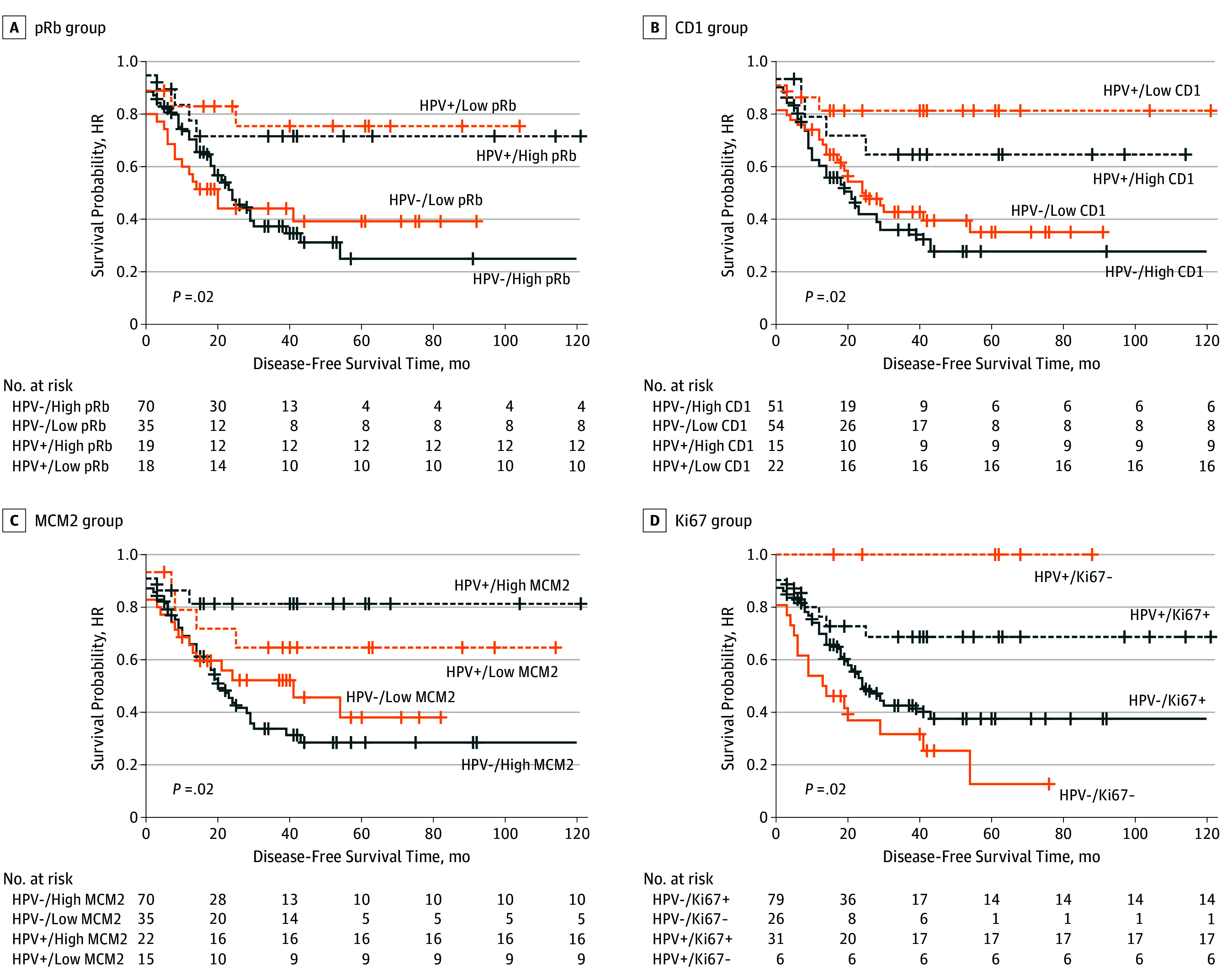
Disease-Free Survival Among Patients With High-Grade Dysplasia or Esophageal Adenocarcinoma as a Function of Human Papillomavirus (HPV)–Stratified Biomarkers Rephrase as follows: Of 142 patients with either high-grade Barrett dysplasia or esophageal adenocarcinoma, 37 were HPV positive and 105 were HPV negative. Of note, HPV positive/low pRb (A), HPV positive/low CD1 (B), HPV positive/high MCM2 (C) and HPV positive/Ki-67 negative (D) were associated with the best disease-free survival. CD1 indicates cyclin D1; HR, hazard ratio; MCM2, minichromosome maintenance protein; and pRb, retinoblastoma protein.

The pRb, CD1, MCM2 and Ki-67 markers stratified by HPV status had a significant association with disease-free survival on univariate analysis. On multivariate analysis, only pRb, CD1 and MCM2 stratified by HPV status maintained a significant association with diseases-free survival (Table 2 and Figure 1). Thus, patients with EAC that was HPV-positive with low pRb expression were associated with significantly improved disease-free survival compared with HPV-negative, high-expression pRb on univariate analysis (HR, 0.29; 95% CI, 0.10-0.82; *P* = .02) and even after adjusting for age, sex, body mass index, ever smoked, excess alcohol use, and resection margin (HR, 0.33; 95% CI, 0.12-0.93; adjusted *P* = .04). In the case of HPV-positive, high-expression pRb, there was improved disease-free survival on univariate analysis, but not after adjustment for confounders (Table 2). Similarly, on multivariate analysis, HPV-positive, low-expression CD1 was associated with a significantly favorable disease-free survival (HR, 0.26; 95% CI, 0.09-0.76; adjusted *P* = .01), as was HPV-positive, high-expression MCM2 (HR, 0.27; 95% CI, 0.09-0.78; adjusted *P* = .02) (Table 2). Human papillomavirus–positive, Ki-67-positive HGD/EAC was associated with improved prognosis on univariate analysis (HR, 0.47; 95% CI, 0.23-0.96; *P* = .04), but not after adjustment for confounders (HR, 0.49; 95% CI, 0.23-1.07; adjusted *P* = .07).

**Table 2.** Log-Rank and Multivariate Disease-Free Survival Analysis of Biomarkers Stratified by HPV Status

Biomarker	Disease-Free Survival			
	Model 1^a^		Model 2^b^	
	HR (95% CI)	Unadjusted P Value	Adjusted HR (95% CI)	Adjusted P Value
**Retinoblastoma Protein**				
HPV-negative				
High expression	1 [Reference]	NA	1 [Reference]	NA
Low expression	1.01 (0.59-1.72)	.98	1.11 (0.62-2.00)	.73
HPV-positive				
High expression	0.37 (0.15-0.94)	.04	0.37 (0.13-1.05)	.06
Low expression	0.29 (0.10-0.82)	.02	0.33 (0.12-0.93)	.04
**Cyclin D1**				
HPV-negative				
High expression	1 [Reference]	NA	1 [Reference]	NA
Low expression	0.83 (0.50-1.37)	.46	0.89 (0.51-1.54)	.67
HPV-positive				
High expression	0.40 (0.16-1.04)	.06	0.41 (0.14-1.22)	.11
Low expression	0.23 (0.08-0.64)	.005	0.26 (0.09-0.76)	.01
**Minichromosome Maintenance Protein 2**				
HPV-negative				
High expression	1 [Reference]	NA	1 [Reference]	NA
Low expression	0.79 (0.46-1.37)	.40	0.94 (0.52-1.69)	.83
HPV-positive				
High expression	0.23 (0.08-0.65)	.005	0.27 (0.09-0.78)	.02
Low expression	0.41 (0.16-1.04)	.06	0.43 (0.15-1.24)	.12
**Ki-67**				
HPV-negative				
Positive	1 [Reference]	NA	1 [Reference]	NA
Negative	1.62 (0.94-2.78)	.08	1.45 (0.79-2.65)	.23
HPV-positive				
Positive	0.47 (0.23-0.96)	.04	0.49 (0.23-1.07)	.07
Negative	NA	NA	NA	NA

Abbreviations: HR, hazard ratio; HPV, human papillomavirus; NA, not applicable.

^a^ Model 1 was a univariate analysis on each characteristic with disease-free survival.

^b^ In model 2, each characteristic was analyzed separately using multivariate Cox proportional hazards regression, adjusted by the following covariates: age, sex, body mass index, ever smoked, excess alcohol use, and R0 resection margin.

**Figure 2.**
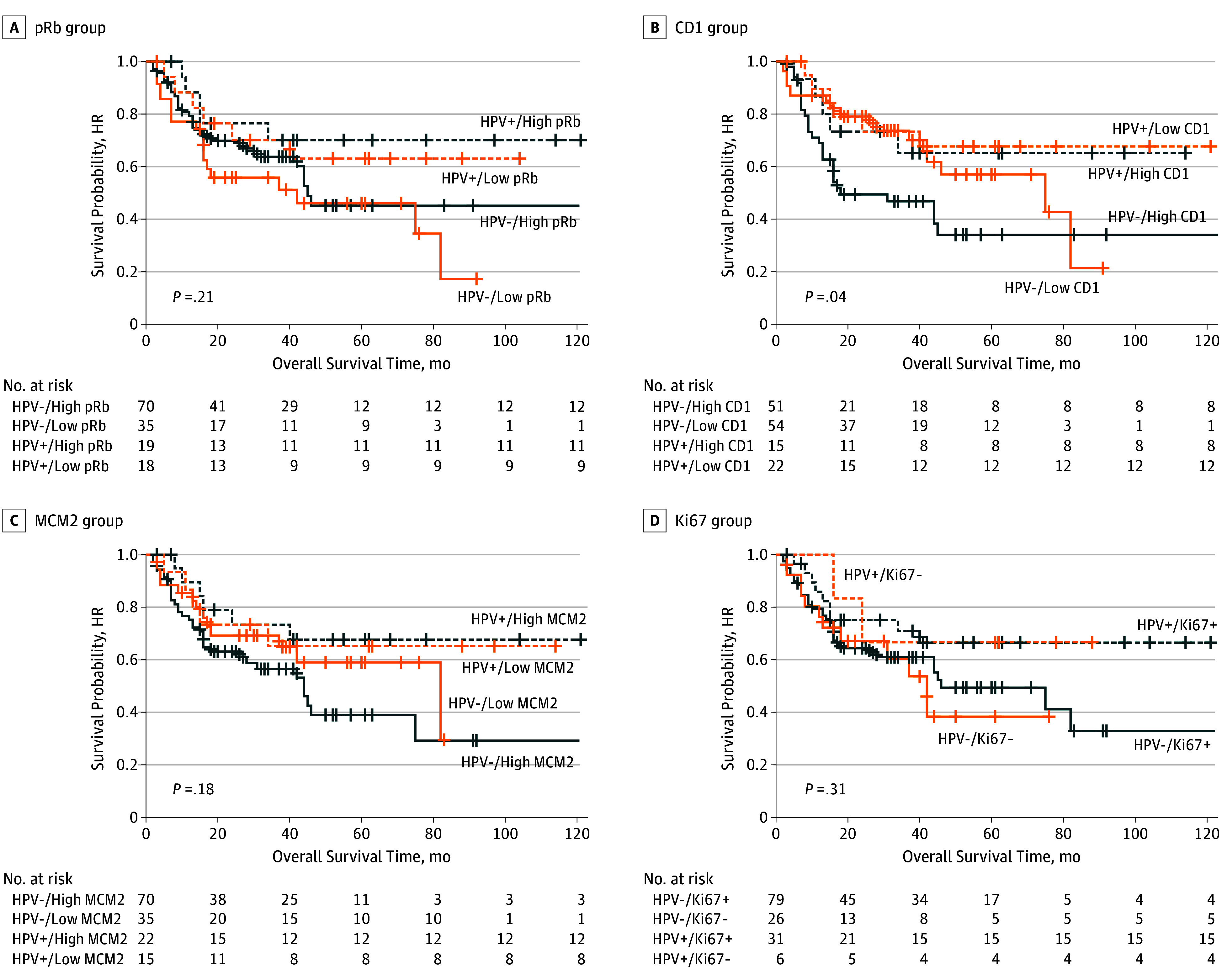
Overall Survival Among Patients With High-Grade Dysplasia or Esophageal Adenocarcinoma as a Function of Human Papillomavirus (HPV)–Stratified Biomarkers Of 142 patients with either high-grade dysplasia or esophageal adenocarcinoma, 37 were HPV positive and 105 were HPV negative. Of note, HPV positive/high pRb (A), HPV positive/low CD1 (B), HPV positive/high MCM2 (C) and HPV positive/Ki-67 negative (D) appeared to be associated with the best overall survival. Nevertheless, only the HPV positive/low CD1 result reached statistical significance. CD1 indicates cyclin D1; HR, hazard ratio; MCM2, minichromosome maintenance protein; and pRb, retinoblastoma protein.

In regard to overall survival, HPV had a significant association only with low expression of CD1, which was associated with significant improvements in survival both on univariate analysis and after adjustment for confounders (HR, 0.38; 95% CI, 0.15-0.94; adjusted *P* = .04) (Table 3 and Figure 2). None of the other biomarkers stratified for HPV status was associated with overall survival.

**Table 3.** Log-Rank and Multivariate Overall Survival Analysis of Biomarkers Stratified by HPV Status

Biomarker	Overall Survival			
	Model 1^a^		Model 2^b^	
	HR (95% CI)	Unadjusted *P* Value	Adjusted HR (95% CI)	Adjusted *P* Value
**Retinoblastoma Protein**				
HPV-negative				
High expression	1 [Reference]	NA	1 [Reference]	NA
Low expression	1.34 (0.75-2.40)	.33	1.78 (0.92-3.44)	.09
HPV-positive				
High expression	0.53 (0.21-1.38)	.20	0.55 (0.19-1.59)	.27
Low expression	0.67 (0.28-1.62)	.37	0.72 (0.29-1.79)	.48
**Cyclin D1**				
HPV-negative				
High expression	1 [Reference]	NA	1 [Reference]	NA
Low expression	0.54 (0.20-0.96)	.03	0.56 (0.29-1.06)	.07
HPV-positive				
High expression	0.43 (0.17-1.13)	.09	0.47 (0.16-1.43)	.18
Low expression	0.37 (0.15-0.89)	.03	0.38 (0.15-0.94)	.04
**Minichromosome Maintenance Protein 2**				
HPV-negative				
High expression	1 [Reference]	NA	1 [Reference]	NA
Low expression	0.69 (0.36-1.31)	.25	0.81 (0.41-1.61)	.55
HPV-positive				
High expression	0.44 (0.19-1.06)	.07	0.45 (0.18-1.12)	.09
Low expression	0.52 (0.20-1.34)	.18	0.60 (0.20-1.75)	.35
**Ki-67**				
HPV-negative				
Positive	1 [Reference]	NA	1 [Reference]	NA
Negative	1.14 (0.59-2.21)	.69	0.79 (0.37-1.67)	.53
HPV-positive				
Positive	0.57 (0.27-1.19)	.13	0.53 (0.24-1.16)	.11
Negative	0.50 (0.12-2.07)	.34	0.41 (0.09-1.78)	.23

Abbreviations: HPV, human papillomavirus; HR, hazard ratio; NA, not applicable.

^a^ Model 1 was a univariate analysis on each characteristic with disease-free survival.

^b^ In model 2, each characteristic was analyzed separately, using multivariate Cox proportional hazards regression, adjusted for the following covariates: age, sex, body mass index, ever smoked, excess alcohol use, and R0 resection margin.

### Biomarkers Stratified by HPV Status and Survival

Human papillomavirus status again had a positive association with CD1, pRb, and MCM2 regarding disease-free survival, disease relapse and progression, and disease-specific death. Human papillomavirus-positive, low- or high-expression pRb Barrett HGD and EAC were associated with substantially improved disease-free survival (41.2 months vs 39.5 months vs 25.5 months vs 23.4 months, respectively; *P* = .03), reduced progression and recurrence (4 patients vs 5 patients vs 20 patients vs 41 patients, respectively; *P* = .006), distant metastases (3 patients vs 0 patients vs 13 patients vs 16 patients, respectively; *P* = .02) and death due to EAC (2 patients vs 3 patients vs 15 patients vs 23 patients, respectively; *P* = .049) compared with HPV-negative, low-or high-expression pRb lesions (eTable 2 in the Supplement). Overall, HPV-positive, low-expression or high-expression CD1 Barrett HGD and EAC lesions were associated with superior disease-free survival (38.9 months vs 42.5 months vs 26.3 months vs 21.7 months, respectively; *P* = .02), reduced recurrence or progression (4 patients vs 5 patients vs 31 patients vs 30 patients, respectively; *P* = .004), distant metastases (2 patients vs 1 patient vs 12 patients vs 17 patients, respectively; *P* = .049) and death due to EAC (2 patients vs 3 patients vs 16 patients vs 22 patients, respectively; *P* = .024) compared with HPV-negative, low-or high-expression CD1 (eTable 3 in the Supplement). Patients with HPV-positive, high-expression MCM2 had better clinical outcomes in terms of recurrence or progression and death due to EAC compared with HPV-negative, high-expression MCM2 (4 patients vs 43 patients; *P* = .003; and 2 patients vs 28 patients; *P* = .035, respectively) (eTable 4 in the Supplement). For Ki-67–negative esophageal tumors, HPV positivity was associated with superior disease-free and overall survival compared with HPV-negative/Ki-67–negative lesions (53.2 months vs 18.8 months; *P* = .009; and 54.8 months vs 25.8 months; *P* = .03, respectively) (eTable 5 in the Supplement).

## Discussion

In this study of 142 patients with Barrett HGD or EAC, we retrospectively examined the prognostic value of cell-cycle markers that have been previously documented to be associated with HPV-associated cancers. This study enabled us to examine how molecular aberrations, clinicopathologic characteristics, and effect size modification of HPV may be associated with survival in this cohort of patients with EAC.

The pRb, CD1, MCM2, and Ki-67 biomarkers were not independently associated with disease-free survival. Regarding overall survival, only low CD1 expression was associated with a significantly improved prognosis in patients with EAC. In this regard, a case-control analysis of CD1 overexpression in Barrett metaplasia has been associated with an increased risk of adenocarcinoma (odds ratio, 6.85; 95% CI, 1.57-29.91; *P* = .01).^36^

Human papillomavirus–positive status was associated with disease-free survival and low-expression pRb, low-expression CD1, and high-expression MCM2. In regard to overall survival, only low expression of CD1 had prognostic significance in Barrett HGD and EAC when stratified according to HPV status. Low expression of pRb, low expression of CD1, and high expression of MCM2 were all associated with superior disease-free survival for HPV-positive tumors compared with HPV negative esophageal lesions. Again, HPV stratification of low expression pRb, low-expression CD1 and high expression MCM2 was significantly associated with outcomes pertaining to survival, disease relapse and progression and disease-specific death.

The potential influence of HPV in association with other biomarkers and survival outcomes has also been described in oropharyngeal squamous cell carcinomas.^37^ Hypoxia-inducible factor-1 transcription factor overexpression predicted worse survival in HPV-positive compared with HPV-negative oropharyngeal head and neck squamous cell carcinomas. Similarly, it has been reported that Ki-67 expression has improved prognostic significance when stratified according to HPV status in HPV-induced head and neck squamous cell carcinomas.^17^ The best survival outcomes were identified in HPV-positive, Ki-67–negative tumors and the worst in HPV-negative, Ki-67–positive HPV-induced head and neck squamous cell carcinomas. In this cohort of patients with HGD/EAC, those with HPV-positive/Ki-67–negative tumors were associated with superior disease-free survival and overall survival compared with individuals possessing HPV-negative/Ki-67–negative esophageal lesions.

Altered MCM2 expression signifies cell-cycle deregulation, which is necessary for the initiation and progression of cancer. Human papillomavirus infection is responsible for overexpression of this protein in cervical dysplasia, with consequent uncontrolled activation of gene transcription and aberrant S-phase induction mediated via the E2F transcription factor pathway.^38^

In our study, patients with HPV-positive, high-expression MCM2 Barrett HGD and EAC were associated with better clinical outcomes in terms of recurrence or progression and death due to EAC compared with HPV-negative, high-expression MCM2. Moreover, on multivariable analysis, HPV-positive/high-expression MCM2 esophageal tumors had a superior disease-free survival as compared with HPV-negative/high-expression MCM2. Minichromosome maintenance protein is considered a surrogate marker of E6 and E7 expression, which are oncoproteins of high-risk HPV.^39,40^ In this regard, a previous study demonstrated improved disease-free survival in transcriptionally active HPV- and E6/E7-associated Barrett HGD and EAC.^11^

Proteolysis with consequent degradation and inactivation of pRb by the HPV E7 oncoprotein causes reduced immunostaining of the protein in tumor tissue sections.^20^ In turn, pRb inactivation causes increased expression of p16^INK4A^. Conversely, CD1 staining is reduced or absent because an intact pRb is necessary for its expression.^41^ Upregulation of p16^INK4A^ and downregulation of pRb are cellular consequences of HPV transformation.^14,24^ Accordingly, CD1 mRNA has been shown to be reduced or absent in HPV-induced oropharyngeal squamous cell carcinomas.^19^ Nevertheless, other investigators have reported that low expression of CD1 is an unreliable marker for HPV-induced cervical and laryngeal tumors.^19,25^ A previous cross-sectional study found no association between HPV status and CD1 expression status in esophageal biopsy specimens from patients representing the Barrett esophagus low- and high-grade dysplasia adenocarcinoma sequence.^14^ Plausible reasons include the fact that most of the dysplastic specimens were low grade and the sample size of the Barrett HGD and EAC cohorts was relatively small.

### Strengths and Limitations

Care was undertaken to minimize cross-contamination of samples as described in the Methods section and thus avoid false-positive HPV DNA detection. Central reporting of slides was undertaken by academic gastrointestinal pathologists, which is an added strength of this study.

The study has limitations. The sample size was small, and the retrospective nature and case-control design of the study introduce biases involving selection, information, observation, and confounding. Recruitment of patients from secondary and tertiary medical centers further exacerbates selection bias; however, the unknown HPV status at the time of enrollment and treatment decision mitigated both selection and observer bias. Blinding the scientists and pathologists to the patients’ clinical, virologic, and biomarker status as well as treatment outcome minimized measurement bias. Confounding was reduced with adjustment for potential confounders in the multivariate analysis.

There also was a problem with tissue sampling. Multiple biopsies from the Barrett HGD and EAC segment were unavailable to assess for circumferential and longitudinal discordance for viral and protein marker detection. Microdissection of tissue specimens may have increased the yield of lesional cells for analysis. Immunohistochemistry analysis is subjective and lacks uniform scoring systems. Some tissue specimens used were obtained more than 10 years before this study, which can result in DNA invalidity. Specifically, given the age of some of the samples, the MY09/MY11 primers used in the nested PCR might not be able to amplify the 450–base pair target area.

## Conclusions

This study’s findings suggest that low expression of CD1 appears to be an independent prognostic marker in Barrett HGD and EAC. Tumor HPV status in combination with pRb, CD1, MCM2, or Ki-67 has a significant association with survival, disease relapse and progression, and disease-specific death. Further confirmatory studies are required before clinical use of CD1 and HPV status as prognostic biomarkers.

If these findings are confirmed by others, then understanding the underlying mechanism that underpins these differential survival outcomes between HPV-positive and HPV-negative Barrett HGD and EAC would be paramount. This understanding could translate into improved treatment selection for these patients based on HPV status.

## Notes

Supplement. **eTable 1.** Demographic and Clinical Characteristics of the Study Population and Associated Tumors According to Patient Group **eFigure 1.** Flow Diagram Showing HPV DNA and Biomarker Status of Enrolled Study Patients **eFigure 2.** Positive Nuclear Staining Patterns for pRb, Cyclin D1, MCM2, Ki-67; Magnification ×20 **eTable 2.** Comparison of Survival, Disease Relapse, Progression and Site of Failure in Low pRb or High pRb Expression Patients Stratified by HPV Status **eTable 3.** Comparison of Survival, Disease Relapse, Progression and Site of Failure in Low CD1 or High CD1 Expression Patients Stratified by HPV Status **eTable 4.** Comparison of Survival, Disease Relapse, Progression and Site of Failure in Low MCM2 or High MCM2 Expression Patients Stratified by HPV Status **eTable 5.** Comparison of Survival, Disease Relapse, Progression and Site of Failure in Ki-67 Positive or Negative Patients Stratified by HPV Status

## References

[1] Rajendra S, Wang B, Snow ET, . Transcriptionally active human papillomavirus is strongly associated with Barrett’s dysplasia and esophageal adenocarcinoma. Am J Gastroenterol. 2013;108(7):1082-1093. doi:10.1038/ajg.2013.94 23588239

[2] Wang B, Rajendra S, Pavey D, . Viral load and integration status of high-risk human papillomaviruses in the Barrett’s metaplasia-dysplasia-adenocarcinoma sequence. Am J Gastroenterol. 2013;108(11):1814-1816. doi:10.1038/ajg.2013.206 24192963

[3] El-Serag HB, Hollier JM, Gravitt P, Alsarraj A, Younes M. Human papillomavirus and the risk of Barrett’s esophagus. Dis Esophagus. 2013;26(5):517-521. doi:10.1111/j.1442-2050.2012.01392.x 22891654 PMC4412355

[4] Rai N, Jenkins GJ, McAdam E, Hibbitts SJ, Fiander AN, Powell NG. Human papillomavirus infection in Barrett’s oesophagus in the UK: an infrequent event. J Clin Virol. 2008;43(2):250-252. doi:10.1016/j.jcv.2008.07.004 18718811

[5] Iyer A, Rajendran V, Adamson CS, Peng Z, Cooper K, Evans MF. Human papillomavirus is detectable in Barrett’s esophagus and esophageal carcinoma but is unlikely to be of any etiologic significance. J Clin Virol. 2011;50(3):205-208. doi:10.1016/j.jcv.2010.11.015 21169053

[6] Rajendra S, Wang B. Human papillomavirus not detected in esophageal adenocarcinoma tumor specimens. Cancer Epidemiol. 2016;43:119. doi:10.1016/j.canep.2016.05.004 27230106

[7] Antonsson A, Knight L, Whiteman DC; Australian Cancer Study. Human papillomavirus not detected in esophageal adenocarcinoma tumor specimens. Cancer Epidemiol. 2016;41:96-98. doi:10.1016/j.canep.2016.01.014 26895084

[8] Li X, Gao C, Yang Y, . Systematic review with meta-analysis: the association between human papillomavirus infection and oesophageal cancer. Aliment Pharmacol Ther. 2014;39(3):270-281. doi:10.1111/apt.12574 24308856

[9] Kunzmann AT, Graham S, McShane CM, . The prevalence of viral agents in esophageal adenocarcinoma and Barrett’s esophagus: a systematic review. Eur J Gastroenterol Hepatol. 2017;29(7):817-825. doi:10.1097/MEG.0000000000000868 28252462

[10] Rajendra S, Wang B, Merrett N, . Genomic analysis of HPV-positive versus HPV-negative oesophageal adenocarcinoma identifies a differential mutational landscape. J Med Genet. 2016;53(4):227-231. doi:10.1136/jmedgenet-2015-103411 26470716

[11] Rajendra S, Xuan W, Merrett N, . Survival rates for patients with Barrett high-grade dysplasia and esophageal adenocarcinoma with or without human papillomavirus infection. JAMA Netw Open. 2018;1(4):e181054. doi:10.1001/jamanetworkopen.2018.1054 30646096 PMC6324261

[12] Fakhry C, Westra WH, Li S, . Improved survival of patients with human papillomavirus–positive head and neck squamous cell carcinoma in a prospective clinical trial. J Natl Cancer Inst. 2008;100(4):261-269. doi:10.1093/jnci/djn011 18270337

[13] Ang KK, Harris J, Wheeler R, . Human papillomavirus and survival of patients with oropharyngeal cancer. N Engl J Med. 2010;363(1):24-35. doi:10.1056/NEJMoa0912217 20530316 PMC2943767

[14] Rajendra S, Yang T, Xuan W, . Active human papillomavirus involvement in Barrett’s dysplasia and oesophageal adenocarcinoma is characterized by wild-type p53 and aberrations of the retinoblastoma protein pathway. Int J Cancer. 2017;141(10):2037-2049. doi:10.1002/ijc.30896 28722212

[15] McLaughlin-Drubin ME, Crum CP, Münger K. Human papillomavirus E7 oncoprotein induces KDM6A and KDM6B histone demethylase expression and causes epigenetic reprogramming. Proc Natl Acad Sci U S A. 2011;108(5):2130-2135. doi:10.1073/pnas.1009933108 21245294 PMC3033314

[16] Doorbar J. Papillomavirus life cycle organization and biomarker selection. Dis Markers. 2007;23(4):297-313. doi:10.1155/2007/613150 17627064 PMC3851388

[17] Liu J, Zhang M, Rose B, . Ki67 Expression has prognostic significance in relation to human papillomavirus status in oropharyngeal squamous cell carcinoma. Ann Surg Oncol. 2015;22(6):1893-1900. doi:10.1245/s10434-014-4237-x 25404475

[18] Boyer SN, Wazer DE, Band V. E7 Protein of human papilloma virus-16 induces degradation of retinoblastoma protein through the ubiquitin-proteasome pathway. Cancer Res. 1996;56(20):4620-4624. 8840974

[19] Andl T, Kahn T, Pfuhl A, . Etiological involvement of oncogenic human papillomavirus in tonsillar squamous cell carcinomas lacking retinoblastoma cell cycle control. Cancer Res. 1998;58(1):5-13. 9426048

[20] Weinberger PM, Yu Z, Haffty BG, . Molecular classification identifies a subset of human papillomavirus–associated oropharyngeal cancers with favorable prognosis. J Clin Oncol. 2006;24(5):736-747. doi:10.1200/JCO.2004.00.3335 16401683

[21] Arber N, Gammon MD, Hibshoosh H, . Overexpression of cyclin D1 occurs in both squamous carcinomas and adenocarcinomas of the esophagus and in adenocarcinomas of the stomach. Hum Pathol. 1999;30(9):1087-1092. doi:10.1016/S0046-8177(99)90227-7 10492044

[22] Langer R, Von Rahden BHA, Nahrig J, . Prognostic significance of expression patterns of c-erbB-2, p53, p16INK4A, p27KIP1, cyclin D1 and epidermal growth factor receptor in oesophageal adenocarcinoma: a tissue microarray study. J Clin Pathol. 2006;59(6):631-634. doi:10.1136/jcp.2005.034298 16731604 PMC1860401

[23] Hong A, Zhang X, Jones D, . Relationships between p53 mutation, HPV status and outcome in oropharyngeal squamous cell carcinoma. Radiother Oncol. 2016;118(2):342-349. doi:10.1016/j.radonc.2016.02.009 26952933

[24] Halec G, Holzinger D, Schmitt M, . Biological evidence for a causal role of HPV16 in a small fraction of laryngeal squamous cell carcinoma. Br J Cancer. 2013;109(1):172-183. doi:10.1038/bjc.2013.296 23778529 PMC3708587

[25] Halec G, Schmitt M, Dondog B, . Biological activity of probable/possible high-risk human papillomavirus types in cervical cancer. Int J Cancer. 2013;132(1):63-71. doi:10.1002/ijc.27605 22514107

[26] Frigola J, Remus D, Mehanna A, Diffley JF. ATPase-dependent quality control of DNA replication origin licensing. Nature. 2013;495(7441):339-343. doi:10.1038/nature11920 23474987 PMC4825857

[27] Kikuchi J, Kinoshita I, Shimizu Y, . Minichromosome maintenance (MCM) protein 4 as a marker for proliferation and its clinical and clinicopathological significance in non–small cell lung cancer. Lung Cancer. 2011;72(2):229-237. doi:10.1016/j.lungcan.2010.08.020 20884074

[28] Going JJ, Keith WN, Neilson L, Stoeber K, Stuart RC, Williams GH. Aberrant expression of minichromosome maintenance proteins 2 and 5, and Ki-67 in dysplastic squamous oesophageal epithelium and Barrett’s mucosa. Gut. 2002;50(3):373-377. doi:10.1136/gut.50.3.373 11839717 PMC1773132

[29] Sirieix PS, O’Donovan M, Brown J, Save V, Coleman N, Fitzgerald RC. Surface expression of minichromosome maintenance proteins provides a novel method for detecting patients at risk for developing adenocarcinoma in Barrett’s esophagus. Clin Cancer Res. 2003;9(7):2560-2566. 12855631

[30] Choy B, LaLonde A, Que J, Wu T, Zhou Z. MCM4 and MCM7, potential novel proliferation markers, significantly correlated with Ki-67, Bmi1, and cyclin E expression in esophageal adenocarcinoma, squamous cell carcinoma, and precancerous lesions. Hum Pathol. 2016;57:126-135. doi:10.1016/j.humpath.2016.07.013 27476776 PMC5250507

[31] Halloush RA, Akpolat I, Jim Zhai Q, Schwartz MR, Mody DR. Comparison of ProEx C with p16INK4a and Ki-67 immunohistochemical staining of cell blocks prepared from residual liquid-based cervicovaginal material: a pilot study. Cancer. 2008;114(6):474-480. doi:10.1002/cncr.23951 19016301

[32] Mukherjee G, Muralidhar B, Bafna UD, Laskey RA, Coleman N. MCM immunocytochemistry as a first line cervical screening test in developing countries: a prospective cohort study in a regional cancer centre in India. Br J Cancer. 2007;96(7):1107-1111. doi:10.1038/sj.bjc.6603679 17342084 PMC2360130

[33] Edge SB, Compton CC. The American Joint Committee on Cancer: the 7th edition of the AJCC cancer staging manual and the future of TNM. Ann Surg Oncol. 2010;17(6):1471-1474. doi:10.1245/s10434-010-0985-4 20180029

[34] Holzinger D, Flechtenmacher C, Henfling N, . Identification of oropharyngeal squamous cell carcinomas with active HPV16 involvement by immunohistochemical analysis of the retinoblastoma protein pathway. Int J Cancer. 2013;133(6):1389-1399. doi:10.1002/ijc.28142 23457055

[35] Hage M, Siersema PD, Vissers KJ, . Molecular evaluation of ablative therapy of Barrett’s oesophagus. J Pathol. 2005;205(1):57-64. doi:10.1002/path.1685 15586364

[36] Bani-Hani K, Martin IG, Hardie LJ, . Prospective study of cyclin D1 overexpression in Barrett’s esophagus: association with increased risk of adenocarcinoma. J Natl Cancer Inst. 2000;92(16):1316-1321. doi:10.1093/jnci/92.16.1316 10944553

[37] Swartz JE, Pothen AJ, van Kempen PM, . Poor prognosis in human papillomavirus-positive oropharyngeal squamous cell carcinomas that overexpress hypoxia inducible factor-1α. Head Neck. 2016;38(9):1338-1346. doi:10.1002/hed.24445 27027530

[38] Ishimi Y, Okayasu I, Kato C, . Enhanced expression of MCM proteins in cancer cells derived from uterine cervix. Eur J Biochem. 2003;270(6):1089-1101. doi:10.1046/j.1432-1033.2003.03440.x 12631269

[39] Maiorano D, Lutzmann M, Méchali M. MCM proteins and DNA replication. Curr Opin Cell Biol. 2006;18(2):130-136. doi:10.1016/j.ceb.2006.02.006 16495042

[40] Murphy N, Ring M, Heffron CC, . Quantitation of CDC6 and MCM5 mRNA in cervical intraepithelial neoplasia and invasive squamous cell carcinoma of the cervix. Mod Pathol. 2005;18(6):844-849. doi:10.1038/modpathol.3800361 15696126

[41] Bates S, Parry D, Bonetta L, Vousden K, Dickson C, Peters G. Absence of cyclin D/cdk complexes in cells lacking functional retinoblastoma protein. Oncogene. 1994;9(6):1633-1640. 8183557

