# Supplementary material for: Association of Biomarkers for Human Papillomavirus With Survival Among Adults With Barrett High-grade Dysplasia and Esophageal Adenocarcinoma
Source: JAMA Netw Open. 2020 Feb 14;3(2):e1921189. doi: 10.1001/jamanetworkopen.2019.21189 (PMC12064093; doi:10.1001/jamanetworkopen.2019.21189)
Supplement: Supplement. — eTable 1. Demographic and Clinical Characteristics of the Study Population and Associated Tumors According to Patient Group eFigure 1. Flow Diagram Showing HPV DNA and Biomarker Status of Enrolled Study Patients eFigure 2. Positive Nuclear Staining Patterns for pRb, Cyclin D1, MCM2, Ki-67; Magnification ×20 eTable 2. Comparison of Survival, Disease Relapse, Progression and Site of Failure in Low pRb or High pRb Expression Patients Stratified by HPV Status eTable 3. Comparison of Survival, Disease Relapse, Progression and Site of Failure in Low CD1 or High CD1 Expression Patients Stratified by HPV Status eTable 4. Comparison of Survival, Disease Relapse, Progression and Site of Failure in Low MCM2 or High MCM2 Expression Patients Stratified by HPV Status eTable 5. Comparison of Survival, Disease Relapse, Progression and Site of Failure in Ki-67 Positive or Negative Patients Stratified by HPV Status [file jamanetwopen-e1921189-s001.pdf]

Supplementary Online Content

Rajendra S, Sharma P, Gautam SD, et al. Association of biomarkers for human papillomavirus with survival among adults with Barrett high-grade dysplasia and esophageal adenocarcinoma. *JAMA Netw Open*. 2020;3(2):e1921189. doi:10.1001/jamanetworkopen.2019.21189

eTable 1. Demographic and Clinical Characteristics of the Study Population and Associated Tumors According to Patient Group

eFigure 1. Flow Diagram Showing HPV DNA and Biomarker Status of Enrolled Study Patients

eFigure 2. Positive Nuclear Staining Patterns for pRb, Cyclin D1, MCM2, Ki-67; Magnification ×20

eTable 2. Comparison of Survival, Disease Relapse, Progression and Site of Failure in Low pRb or High pRb Expression Patients Stratified by HPV Status

eTable 3. Comparison of Survival, Disease Relapse, Progression and Site of Failure in Low CD1 or High CD1 Expression Patients Stratified by HPV Status

eTable 4. Comparison of Survival, Disease Relapse, Progression and Site of Failure in Low MCM2 or High MCM2 Expression Patients Stratified by HPV Status

eTable 5. Comparison of Survival, Disease Relapse, Progression and Site of Failure in Ki-67 Positive or Negative Patients Stratified by HPV Status

This supplementary material has been provided by the authors to give readers additional information about their work.

© 2020 Rajendra S et al. *JAMA Network Open.*

eTable 1. Demographic and clinical characteristics of the study population and associated tumors according to patient group.

Characteristic	HPV Positive HGD/EAC patients (n=37, 26.1%)	HPV Negative HGD/EAC patients (n=105, 73.9%)	P-value [a]
**Sex (M/F)**	33 (89.2%)<br>4 (10.80%)	93 (88.6%)<br>12 (11.4%)	0.92
**Age, mean (SD) in years**<br>**Range**	65.2 (SD +/- 12.4)<br>Min=33.0, Max=89.0	66.2 (SD +/- 12.0)<br>Min=32.0, Max=90.0	0.65
**BMI, mean (SD)**	27.7 (SD+/-5.7)	27.0 (SD+/-5.1)	0.53
**Ever smoked**	24/37 (64.9%)	77/105 (73.3%)	0.33
**>10 pack years**[b]	22/24 (91.7%)	69/76 (90.8%)	0.90
**>20 pack years**[b]	15/24 (62.5%)	48/76 (63.2%)	0.95
**Alcohol intake**	26/36 (72.2%)	75/105 (71.4%)	0.93
**Excess Alcohol**[c]	8/35 (22.9%)	18/105 (17.1%)	0.45
**Low pRb**	18/37 (48.7%)	35/105 (33.3%)	0.10
**Low CD1**	22/37 (59.5%)	54/105 (51.4%)	0.40
**Ki-67 positive**	31/37 (83.8%)	79/105 (75.2%)	0.28

© 2020 Rajendra S et al. *JAMA Network Open.*

**High MCM2**	15/37 (40.5%)	70/105 (66.7%)	0.005

[a] Differences between HPV positive versus HPV negative cases regarding baseline characteristics were assessed using two-sample t-test for all numerical data and chi-square analysis for binary measurements.
[b] A pack-year=smoking one packet of cigarettes per day for a year.
[c] Excess alcohol intake=for men >21 units per week, for women >14 units per week.

Abbreviations: BMI; body mass index, CD1; cyclin D1, EAC; esophageal adenocarcinoma,
EMR; endoscopic mucosal resection, HGD; high-grade dysplasia, HPV; human papillomavirus, MCM; minichromosome maintenance protein, PPI; proton-pump inhibitors, pRb; retinoblastoma protein, NSAID's; non-steroidal anti-inflammatory drugs, RFA; radiofrequency ablation, SD; standard deviation

© 2020 Rajendra S et al. *JAMA Network Open.*

eFigure 1. Flow diagram showing HPV DNA and biomarker status of enrolled study patients.

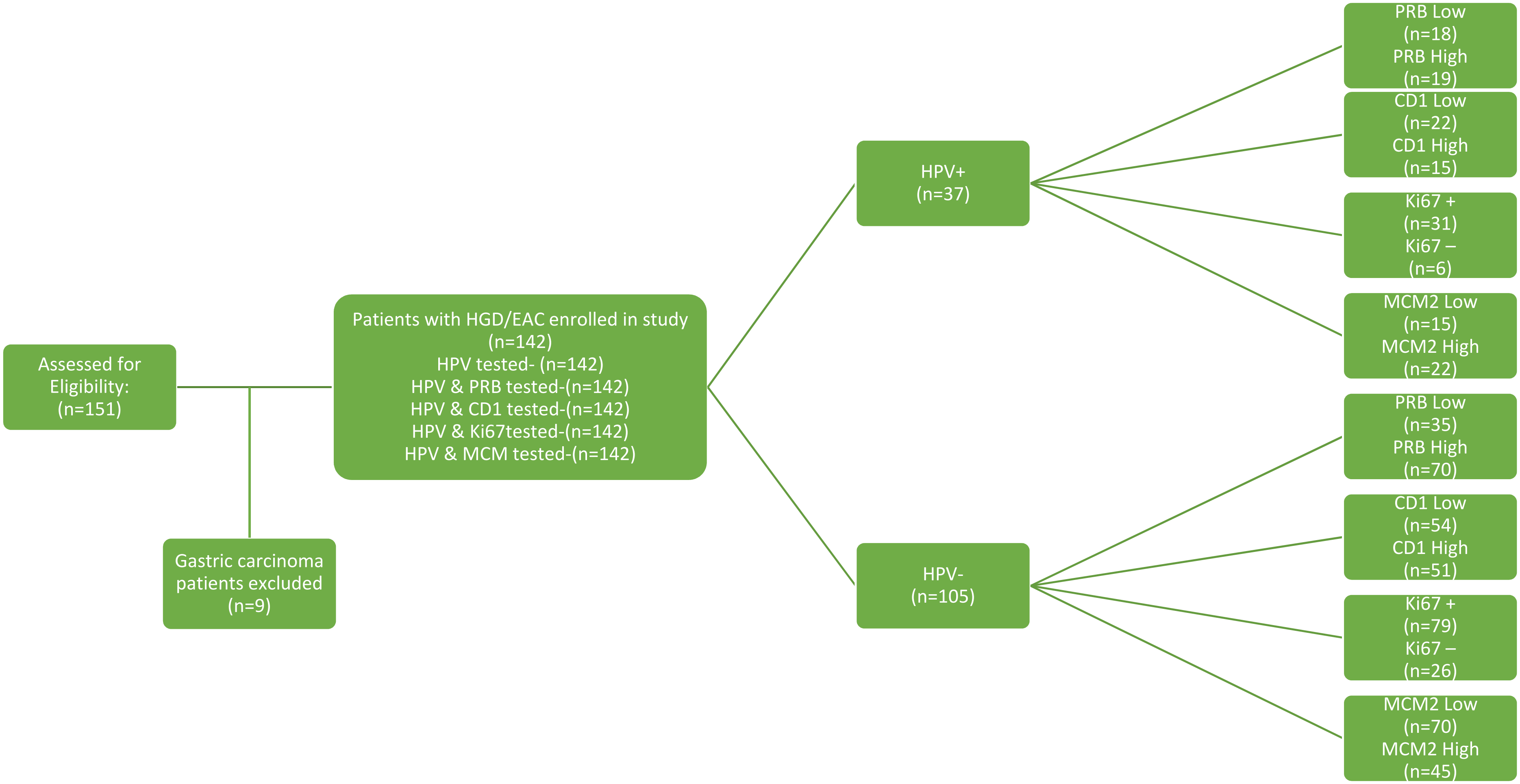

© 2020 Rajendra S et al. *JAMA Network Open.*

eFigure 2. Positive nuclear staining patterns for A) pRb, B) Cyclin D1, C) MCM2, D) Ki-67. Magnification X 20

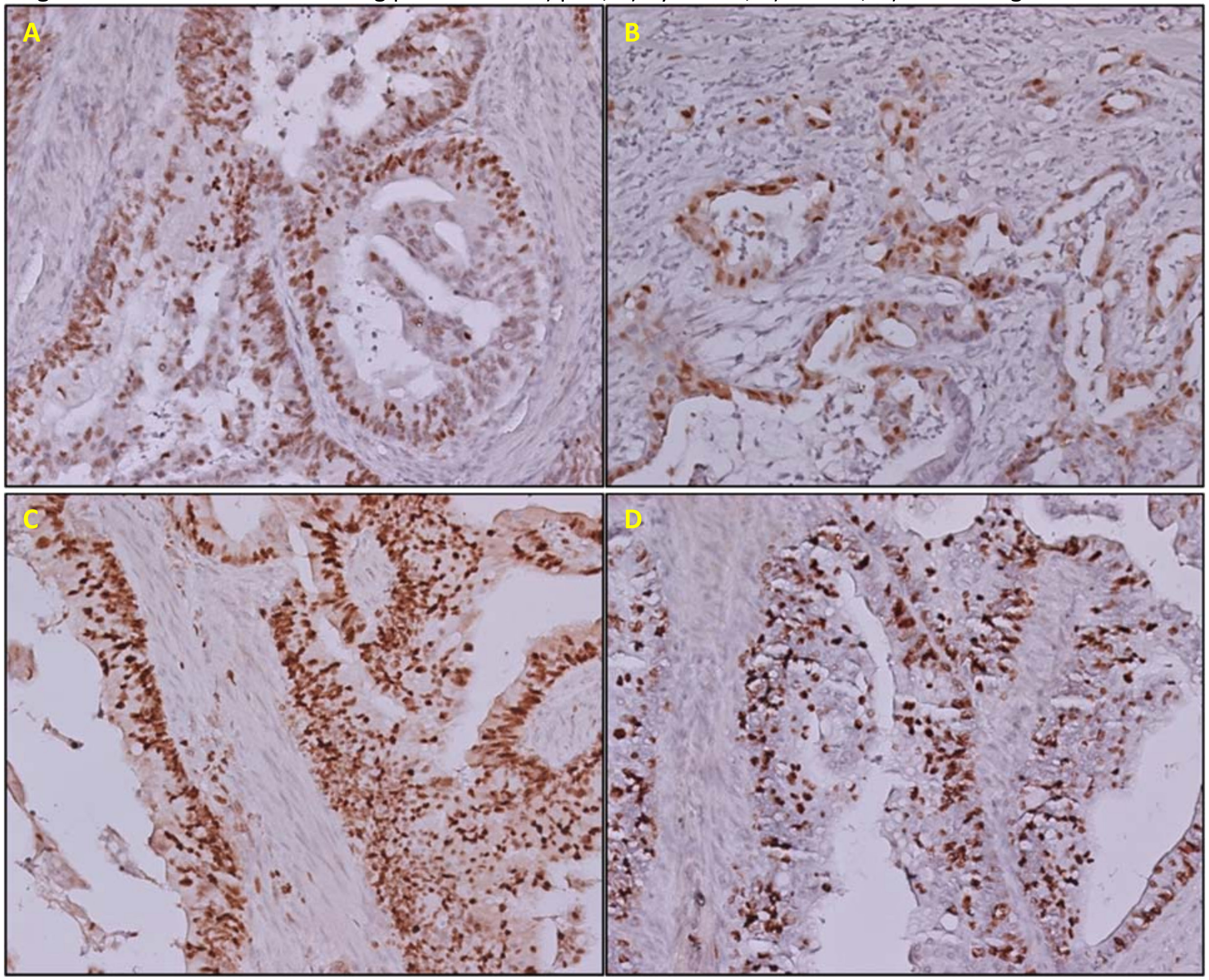

© 2020 Rajendra S et al. *JAMA Network Open.*

eTable 2. Comparison of Survival, Disease Relapse, Progression and Site of Failure in low pRb or high pRb expression patients stratified by HPV status.

	HPV + No. (%CI)		HPV- No. (%CI)		
	**Low pRb (4)**	**High pRb (3)**	**Low pRb (2)**	**High pRb (1)**	**p-value**
**Disease-free survival, mean (SD), mo**	41.2 (31.2)	39.5 (37.0)	25.5 (27.6)	23.4 (24.6)	0.03
**Overall survival, mean (SD), mo**	42.9 (30.5)	44.5 (35.8)	30.2 (25.9)	29.6 (25.2)	0.08
**Survival status (alive at last follow-up)**	12(66.7)	14 (73.7)	16 (45.7)	42 (60.0)	0.19
**Recurrence or progression**	4 (22.2)	5 (26.3)	20 (57.1)	41 (58.6)	0.006
**Recurrence**	2 (11.1)	4 (21.1)	13 (37.1)	33 (47.1)	0.02
**Local-regional failure**	1 (5.6)	5 (26.3)	7 (20.0)	25 (35.7)	0.05
**Distant metastases**	3 (16.7)	0 (0)	13 (37.1)	16 (22.9)	0.02
**Death due to OAC**	2 (11.1)	3 (15.8)	15 (42.9)	23 (32.9)	0.049

Abbreviations: EAC; esophageal adenocarcinoma, HPV; human papillomavirus, mo; months N/A; not applicable, pRb; retinoblastoma protein, SD; standard deviation.

© 2020 Rajendra S et al. *JAMA Network Open.*

eTable 3. Comparison of Survival, Disease Relapse, Progression and Site of Failure in low CD1 or high CD1 expression patients stratified by HPV status

	HPV + No. (%CI)		HPV- No. (%CI)		
	**Low CD1 (4)**	**High CD1 (3)**	**Low CD1 (2)**	**High CD1 (1)**	**p-value**
**Disease-free survival, mean (SD), mo**	38.9 (33.2)	42.5 (35.7)	26.3 (24.0)	21.7 (27.0)	0.02
**Overall survival, mean, mo**	43.1 (33.1)	44.6 (33.6)	32.3 (22.2)	27.1 (28.2)	0.05
**Survival status (alive at last follow-up)**	16 (72.7)	10 (66.7)	35 (64.8)	23 (45.1)	0.07
**Recurrence or progression**	4 (18.2)	5 (33.3)	31 (57.4)	30 (58.8)	0.004
**Recurrence**	2 (9.1)	4 (26.7)	21 (38.9)	25 (49.0)	0.01
**Local-regional failure**	2 (9.1)	4 (26.7)	19 (35.2)	13 (25.5)	0.14
**Distant metastases**	2 (9.1)	1 (6.7)	12 (22.2)	17 (33.3)	0.049
**Death due to OAC**	2 (9.1)	3 (20.0)	16 (29.6)	22 (43.1)	0.024

Abbreviations: EAC; esophageal adenocarcinoma, HPV; human papillomavirus, mo; months N/A; not applicable, CD1; Cyclin D1, SD; standard deviation

© 2020 Rajendra S et al. *JAMA Network Open.*

eTable 4. Comparison of Survival, Disease Relapse, Progression and Site of Failure in low MCM2 or high MCM2 expression patients stratified by HPV status

	HPV + No. (%CI)		HPV- No. (%CI)		
	**Low MCM2 (4)**	**High MCM2 (3)**	**Low MCM2 (2)**	**High MCM2 (1)**	**p-value**
**Disease-free survival, mean (SD), mo**	42.5 (35.7)	38.9 (33.2)	25.3 (23.6)	23.5 (26.5)	0.03
**Overall survival, mean, mo**	44.6 (33.6)	43.1 (33.1)	32.8 (24.0)	28.3 (26.0)	0.06
**Survival status (alive at last follow-up)**	10 (66.7)	16 (72.7)	22 (62.9)	36 (51.4)	0.27
**Recurrence or progression**	5 (33.3)	4 (18.2)	18 (51.4)	43 (61.4)	0.003
**Recurrence**	4 (26.7)	2 (9.1)	12 (34.3)	34 (48.6)	0.007
**Local-regional failure**	4 (26.7)	2 (9.1)	10 (28.6)	22 (31.4)	0.23
**Distant metastases**	1 (6.7)	2 (9.1)	8 (22.9)	21 (30.0)	0.08
**Death due to OAC**	3 (2.5)	2 (9.1)	10 (28.6)	28 (40.0)	0.035

Abbreviations: EAC; esophageal adenocarcinoma, HPV; human papillomavirus, mo; months N/A; not applicable, MCM; Minichromosomal maintenance protein SD; standard deviation.

© 2020 Rajendra S et al. *JAMA Network Open.*

eTable 5. Comparison of Survival, Disease Relapse, Progression and Site of Failure in Ki-67 positive or negative patients stratified by HPV status

	**HPV + No. (%CI)**		**HPV- No. (%CI)**		
	**Ki67 + (4)**	**Ki67 – (3)**	**Ki67 + (2)**	**Ki67 – (1)**	**p-value**
**Disease-free survival, mean (SD), mo**	37.6 (34.7)	53.2 (27.6)	25.9 (27.0)	18.8 (19.7)	0.009
**Overall survival, mean, mo**	41.6 (33.6)	54.8 (28.9)	31.1 (26.9)	25.8 (19.3)	0.03
**Survival status (alive at last follow-up)**	22 (71.0)	4 (66.7)	44 (55.7)	14 (53.9)	0.45
**Recurrence or progression**	9 (29.0)	0 (N/A)	42 (53.2)	19 (73.1)	0.001
**Recurrence**	6 (19.4)	0 (N/A)	32 (40.5)	14 (53.9)	0.01
**Local-regional failure**	6 (19.4)	0 (N/A)	19 (24.1)	13 (50.0)	0.01
**Distant metastases**	3 (9.7)	0 (N/A)	23 (29.1)	6 (23.1)	0.08
**Death due to OAC**	5 (16.1)	0 (N/A)	27 (34.2)	11 (42.3)	0.048

Abbreviations: EAC; esophageal adenocarcinoma, HPV; human papillomavirus, mo; months N/A; not applicable, SD; standard deviation.

© 2020 Rajendra S et al. *JAMA Network Open.*
